# Impaired vascular endothelial function as a perioperative risk predictor – a prospective observational trial

**DOI:** 10.1186/s12871-021-01400-y

**Published:** 2021-07-15

**Authors:** Volker Schick, Marc Boensch, Milan van Edig, Jonas Alfitian, Tülay Pola, Hannes Ecker, Falko Lindacher, Kija Shah-Hosseini, Wolfgang A. Wetsch, Bernhard Riedel, Robert Schier

**Affiliations:** 1 Department of Anesthesiology and Intensive Care Medicine, University of Cologne, Faculty of Medicine and University Hospital Cologne, Kerpener Straße 62, 50937 Cologne, Germany; 2 Institute of Medical Statistics and Computational Biology, Faculty of Medicine, University of Cologne, Cologne, Germany; 3 Department of Anaesthesia, Perioperative and Pain Medicine, Peter MacCallum Cancer Centre and The University of Melbourne, Melbourne, Australia

**Keywords:** Impaired vascular endothelial function, Flow mediated vasodilation, Perioperative risk prediction

## Abstract

**Background:**

In the recent years, an increasing number of patients with multiple comorbidities (e.g. coronary artery disease, diabetes, hypertension) presents to the operating room. The clinical risk factors are accompanied by underlying vascular-endothelial dysfunction, which impairs microcirculation and may predispose to end-organ dysfunction and impaired postoperative outcome. Whether preoperative endothelial dysfunction identifies patients at risk of postoperative complications remains unclear. In this prospective observational study, we tested the hypothesis that impaired flow-mediated dilation (FMD), a non-invasive surrogate marker of endothelial function, correlates with Days at Home within 30 days after surgery (DAH30). DAH30 is a patient-centric metric that captures postoperative complications and importantly also hospital re-admissions.

**Methods:**

Seventy-one patients scheduled for major abdominal surgery were enrolled. FMD was performed pre-operatively prior to major abdominal surgery and patients were dichotomised at a threshold value of 10%. FMD was then correlated with DAH30 (primary endpoint) and postoperative complications (secondary endpoints).

**Results:**

DAH30 did not differ between patients with reduced FMD and normal FMD (14 (4) (median (IQR)) vs. 15 (8), *P* = 0.8). Similary, no differences between both groups were found for CCI (normal FMD: 21 (30) (median (IQR)), reduced FMD: 26 (38), *P* = 0.4) or frequency of major complications (normal FMD: 7 (19%) (n (%)), reduced FMD: 12 (35%), *P* = 0.12). The regression analyses revealed that FMD in combination with ASA status and surgery duration had no additional significant predictive effect for DAH30, CCI or Clavien-Dindo score.

**Conclusion:**

FMD does not add predictive value with regards to DAH30, CCI or Clavien-Dindo score within our study cohort of patients undergoing abdominal surgery.

**Trial registration:**

The study was registered in the German Clinical Trials Register (DRKS00005472), prospectively registered on 25/11/2013.

## Background

An increasing incidence of cardiovascular disease calls for cost-effective, non-invasive point-of-care screening methods to assess vascular risk profile, especially underlying endothelial dysfunction. Brachial artery reactivity testing (BART) allows such assessment by testing the functional vasodilatory response of the endothelium to shear stress of increased blood flow. Here the change in vessel diameter is measured in the vasodilation (reactive hyperemia that is endothelial dependent) that follows 3-min of brachial artery occlusion (using suprasystolic cuff pressure). It generates flow mediated dilation (FMD) that is expressed as the percentage change in vessel diameter relative to baseline vessel diameter (pre-ischaemic) [1]. Impaired FMD has been found to correlate with an increased risk for cardiovascular events [2–4] and can predict progression of vascular disease [5]. Furthermore, diabetic patients with low FMD identifies patients with poor microvascular endothelial function and associates with increased microalbuminuria [6].

Endothelial dysfunction may also contribute to perioperative risk where impaired end-organ perfusion may contribute to postoperative complications. In an early stage, patients with endothelial dysfunction may present with subclinical vascular impairment and it is important to identify these patients preoperatively in order to optimize the perioperative management. Several observational studies have reported that impaired endothelial function associates with postoperative complications [7–10].

We sought to understand the impact of endothelial dysfunction, as measured by BART derived FMD, on the extended postoperative period using a more patient-centric endpoint using Days at Home within 30 days after surgery (DAH30). This parameter considers hospital re-admission rates and has prognostic significance towards survival [11]. DAH30 is a sensitive quality improvement metric that contrasts the usual endpoints of current FMD studies (postoperative complications, length of ICU and hospital stay) that are affected by many factors and require large sample sizes. Therefore, we tested the hypothesis that low FMD values associate with decreased DAH30.

## Materials and methods

### Subjects

This study was approved by the institutional ethics review committee at the University of Cologne, Germany (No. 13–112, Head: Prof. Dr. Drzezga) and conduted in accordance with the ethical principles of the declaration of Helsinki. The study was registered in the German Clinical Trials Register (DRKS00005472) and executed in accordance with the CONSORT statement. After obtaining written informed consent, a total of 83 patients were enrolled in the study. FMD measurements were performed during the pre-anesthetic consultation at the University Hospital of Cologne. Eligibility included adult patients scheduled for major abdominal surgery. Exclusion criteria were inoperability, poor physical function status (Metabolic equivalent of Task [MET] < 4) or conditions making FMD measurement impossible (forearm arteriovenous shunt, lymphatic oedema, open wounds). General demographic data, general comorbidity (Charlson Comorbidity Index), cardiovascular specific comorbidity (revised Cardiac Risk Index; rCRI) and surgical risk scores (Surgical Outcome Risk Tool; SORT) were recorded preoperatively [12–14].

### Study endpoints

The primary study endpoint explored the association of FMD with DAH30, as measured by the quantity of postoperative days at home within 30 days after surgery [11]. Secondary study endpoints included postoperative complications and were assessed on postoperative days 3, 5, 8, 15 and 30 by Clavien-Dindo score and CCI. Specifically, we investigated whether FMD provided additional prognostic value alongside other perioperative risk factors.

### Flow mediated dilation

FMD analysis was performed according to the guidelines for the ultrasound assessment of endothelial-dependent flow-mediated vasodilation of the brachial artery [15]. Patients were positioned supine in a quiet room, with a resting period preceding the test. FMD was measured with the patient’s arm in a comfortable position for imaging of the brachial artery and the positioning was the same among all study patients.

All measurements were performed using a *SonixGPS®* ultrasound device (Ultrasonix, Canada). FMD analysis was conducted according to the technique described by Corretti et al. [16], as follows:

- End-diastolic measurement of baseline brachial artery diameter, using longitudinal sonographic imaging above the antecubital fossa.
- Inflation of a cuff placed on the upper arm, maintaining a pressure of at least 50 mmHg above systolic blood pressure for 3 min.
- Measurement of the brachial artery diameter within 45 s after release of the blood pressure cuff, using the same sonographic technique, to calculate percentage increase in vessel diameter due to flow mediated dilation.

FMD was regarded both as an absolute value and also dichotomized within our analysis. We considered a FMD < 10% as reduced, which was already presented as a suitable threshold by Kuven et al. [17].

### Assessement of postoperative outcome

The postoperative course of patients was observed using a standardized questionnaire, by patient interview, as well as by chart review. Complications were assessed according to the Clavien-Dindo (CD) score [18] and the Comprehensive Complication Index (CCI) [19]. Additionally, complications were dichotomised into major and minor complications based on the threshold of CD ≥ IIIa, which was defined a priori and distinguishes between the requirement of non-invasive versus interventional/surgical treatment. The highest complication score during the 30 days postoperative follow-up period was considered.

### Statistical analysis

Statistical analysis was performed using SPSS 25 (IBM Corp. Armonk, NY, USA) and R version 4.0.3 in cooperation with the Institute of Medical Statistics and Computational Biology, University of Cologne. Statistical power was calculated for a sample size of 71 patients and 37% chance for major postoperative complications and consequent lower DAH30, with a power of 80%, α of 0.05 and a β of 0.2. This assumption was based on the data from our pilot study, where a total of 63 patients was sufficient to demonstrate a cutpoint of FMD < 11.5% associated with a higher incidence of postoperative complications and longer ICU/hospital stays [10].

Normality of data was assessed by Shapiro-Wilk test. Normally distributed variables are reported as mean (standdard deviation (SD)), non-normally distributed variables as median (interquartile range (IQR)), respectively. Categorical data is represented as frequency (percentage). Comparison of central tendencies between two groups was made by the Wilcoxon rank sum test for non-normally distributed data. For normally distributed data, Welch’s t test was applied. Categorical variables were tested by the χ^2^ test or by Fisher’s exact test for small sample sizes.

To estimate the predictive impact of FMD on the postoperative outcome, we performed multivariable regression analyses for both the primary (linear regression) and secondary endpoints (linear regression for CCI, ordinal logistic regression for Clavien-Dindo score). ASA status, surgery duration, and surgery type (oesophagectomy versus non-oesophagectomy) were included as covariates.

## Results

### Demographic and clinical characteristics

A total of 83 patients were enrolled in this study. In ten patients, the planned surgery was cancelled preoperatively and in one patient intraoperatively due to inoperability, while one patient was lost to follow-up in the postoperative period. Therefore, 71 patients completed the study and were included in the data analysis. The demographic and baseline clinical characteristics of the enrolled patients, divided into two groups according to the dichotomised FMD, are reported in Table 1. Patients with reduced FMD were found to have a higher mean BMI (normal FMD: 24.6 (4.1) kg/m^2^ (mean (SD)), reduced FMD: 26.9 (3.8) kg/m^2^, *P* = 0.016). Moreover, individuals exhibited a more frequent intake of AT1 antagonists, when they had a reduced FMD (normal FMD: 2 (5.4%) (n(%)), reduced FMD: 8 (24%), *P* = 0.041). No difference was observed between the central tendencies among the remaining variables. In particular, preoperative risk scores, comorbidities, duration of surgery and type of surgery did not differ between individuals with normal and reduced FMD.

**Table 1:** Characteristics of the study population. Patients were dichotomized according to the FMD

			Flow Mediated Dilation		
Variable	Statistic	Overall, ***N*** = 71	Reduced (< 10%), ***N*** = 34	Normal (≥10%), ***N*** = 37	***P***-Value^**1**^
**Age (years)**	Mean (SD)	64 (11)	63 (10)	64 (12)	0.6
**Sex**					0.059
Female	n (%)	20 (28%)	6 (18%)	14 (38%)	
Male	n (%)	51 (72%)	28 (82%)	23 (62%)	
**ASA**					0.9
1	n (%)	1 (1.4%)	0 (0%)	1 (2.7%)	
2	n (%)	54 (76%)	27 (79%)	27 (73%)	
3	n (%)	16 (23%)	7 (21%)	9 (24%)	
**BMI (kg/m**^**2**^**)**	Mean (SD)	25.7 (4.1)	26.9 (3.8)	24.6 (4.1)	**0.016**
**Smoking**	n (%)	30 (42%)	16 (47%)	14 (38%)	0.4
**Charlson Comorbidity index**	Median (IQR)	2.00 (1.00)	2.00 (1.00)	2.00 (1.00)	0.2
**SORT score**	Median (IQR)	1.48 (1.69)	1.48 (1.69)	1.48 (1.69)	> 0.9
**rCRI ≥ 2**	n (%)	39 (55%)	20 (59%)	19 (51%)	0.5
**Hypertension**	n (%)	33 (46%)	18 (53%)	15 (41%)	0.3
**Coronary heart disease**	n (%)	7 (9.9%)	3 (8.8%)	4 (11%)	> 0.9
**Dyslipidemia**	n (%)	9 (13%)	3 (8.8%)	6 (16%)	0.5
**ACE inihibtor intake**	n (%)	12 (17%)	4 (12%)	8 (22%)	0.3
**AT1 antagonist intake**	n (%)	10 (14%)	8 (24%)	2 (5.4%)	**0.041**
**Statine intake**	n (%)	12 (17%)	8 (24%)	4 (11%)	0.2
**Surgery duration (min)**	Mean (SD)	301 (104)	320 (98)	284 (106)	0.14
**Surgery type**					0.11
Non-Oesophagectomy	n (%)	32 (45%)	12 (35%)	20 (54%)	
Oesophagectomy	n (%)	39 (55%)	22 (65%)	17 (46%)	
**DAH30**	Median (IQR)	14 (6)	14 (4)	15 (8)	0.8
**CCI**	Median (IQR)	21 (36)	26 (38)	21 (30)	0.4
**Clavien-Dindo score**					0.3
0	n (%)	20 (28%)	10 (29%)	10 (27%)	
I	n (%)	11 (15%)	4 (12%)	7 (19%)	
II	n (%)	21 (30%)	8 (24%)	13 (35%)	
IIIa	n (%)	6 (8.5%)	3 (8.8%)	3 (8.1%)	
IIIb	n (%)	8 (11%)	7 (21%)	1 (2.7%)	
IVa	n (%)	3 (4.2%)	1 (2.9%)	2 (5.4%)	
IVb	n (%)	2 (2.8%)	1 (2.9%)	1 (2.7%)	
V	n (%)	0 (0%)	0 (0%)	0 (0%)	
**Complications**					0.12
Major (CD ≥ IIIa)	n (%)	19 (27%)	12 (35%)	7 (19%)	
Minor (CD < IIIa)	n (%)	52 (73%)	22 (65%)	30 (81%)	
**FMD (%)**	Median (IQR)	10 (8)	7 (4)	15 (11)	**< 0.001**

^1^Two Sample t-test; Pearson’s Chi-squared test; Fisher’s exact test; Wilcoxon rank sum test; Wilcoxon rank sum exact test

### FMD characteristics across the study population

For the entire study cohort the median FMD was 10.3% (IQR = 8.4%). Patients were dichotomized according to their individual FMD value as described above. Consequently, 34 patients were assigned to the reduced FMD group, while 37 patients had a normal FMD. Following dichotomisation, the median FMD differed significantly between both groups (normal FMD: 15 (11)% (median (IQR)), reduced FMD: 7 (4)%, *P* < 0.001), indicating an appropriate threshold level. The demographic and clinical characteristics of the patient subgroups according to their FMD group are reported in Table 1.

### Primary endpoint DAH30

**Fig. 1:**
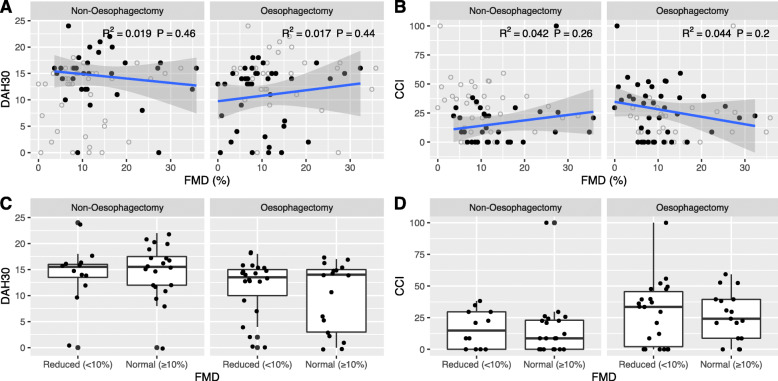
Data of DAH30 and CCI against absolute FMD grouped by surgery type. **A**-**B** Black circles represent the measured data within the respective group, while data of the total group is depicted by unfilled circles. Linear regression was performed and the resulting line (blue) is represented together with its 95% confidence interval (grey area). No significant prediction can be made by the absolute FMD as demonstrated by the *R*^2^ and *P* values. **C**-**D** Central tendencies of DAH30 and CCI were compared between patients with reduced versus normal FMD. In all surgery types, no significant differences could be obtained

DAH30 was the primary endpoint of our study as an indicator of the postoperative outcome. Median DAH30 within the study population was 14 (IQR = 6). After subdivision following dichotomisation by FMD, median DAH30 did not differ between both groups (Table 1). To evaluate whether absolute FMD as an independent variable predicted DAH30, we explored this relationship through linear regression analysis. We additionally examined these relationships after subgrouping by surgery type in view of oesophagectomy as a high-risk operation and therefore possible confounder. However, univariable linear regression analysis for the entire cohort (*R*^2^ = 0.01, *P* = 0.52) as well as following subgrouping (oesophagectomy: *R*^2^ 0.02, *P* = 0.44, non-oesophagectomy: *R*^2^ = 0.02, *P* = 0.46) failed to confirm a significant role of FMD as a predictor of DAH30 (Fig. 1). To account for potential covariates affecting predictability of DAH30, we also performed a multivariable linear regression analysis adjusting for ASA status as well as surgery duration and surgery type as clinically likely predictors alongside FMD as independent variables. Here, ASA status and surgery duration were identified as significant predictors, each with negative coefficients that are clinically plausible (ASA: β = − 4.3, *P* = 0.005, surgery duration: β = − 0.02, *P* = 0.009). However, neither surgery type nor FMD contributed to this regression model to any significant extent (Table 2).

**Table 2:** Results of the multivariable regression analyses for prediction of the outcome variables by FMD

	DAH30^**a**^			CCI^**b**^			Clavien-Dindo		
Predictor	β^**1**^	95% CI^**2**^	***P***-Value	β^**1**^	95% CI^**2**^	***P***-Value	OR^**3**^	95% CI^**2**^	***P***-Value
**FMD (%)**	0.00	−0.16, 0.16	> 0.9	−0.05	−0.67, 0.57	0.9	1.00	0.95, 1.05	0.9
**ASA**	−4.3	−7.2, −1.3	**0.005**	14	2.4, 25	**0.018**	3.78	1.38, 10.9	**0.009**
**Surgery duration (min)**	− 0.02	− 0.03, − 0.01	**0.009**	0.05	0.00, 0.11	0.063	1.01	1.00, 1.01	**0.031**
**Surgery Type**			0.2			0.2			0.14
Non-Oesophagectomy	–	–		–	–		–	–	
Oesophagectomy	−2.0	−5.0, 1.0		7.4	−4.1, 19		2.07	0.79, 5.50	

^1^β = coefficient estimate, multivariable linear regression

^2^CI = Confidence Interval

^3^OR = Odds Ratio, multivariable ordinal logistic regression

^a^*R*^2^ = 0.241

^b^*R*^2^ = 0.171

### Secondary endpoints CCI and Clavien-Dindo score

The comparison of the central tendencies of the CCI between the patients with a normal FMD score and those with reduced FMD did not reveal a significant difference (normal FMD: 21 (30) (median (IQR)), reduced FMD: 26 (38), *P* = 0.4), as demonstrated in Table 1. FMD did not predict CCI in either the univariable regression model (Fig. 1) or the adjusted multivariable regression model, whilst ASA status did (Table 2).

As determined by Clavien-Dindo score, the number of patients suffering no or minor postoperative complications among the whole study cohort was 52 (73%), while 19 (27%) had major complications. However, χ^2^ did not reveal significant indepenence of the subgroups. This finding also emerged upon comparison of the Clavien-Dindo score without dichotomisation. FMD was evaluated as a predictor of Clavien-Dindo score within a multivariable ordinal logistic regression analysis. The resulting regression model was able to predict the Clavien-Dindo score including the independent variables ASA status (OR = 3.78, *P* = 0.009) and surgery duration (OR = 1.01 per minute, *P* = 0.031), however, FMD was obsolete and did not hold significance as a predictor (Table 2).

## Discussion

Poor preoperative (or postoperative decline [8]) of endothelial function may contribute to postoperative complications [7, 20]. Early identification of underlying endothelial dysfunction in the preoperative phase may provide a window of opportunity for preoperative optimization to improve postoperative outcomes. Non-invasive techniques to assess endothelial function are predominantly used for the surveillance of atherosclerosis progression in patients with cardiovascular risk factors. Whether preoperative assessment of non-invasive parameters, e.g. flow mediated dilation,has perioperative utility remains unclear.

In this study cohort, the median recorded FMD value was similar to the values obtained in our pilot study of thoracic surgery patients [10] indicating a comparable measurement technique. However, FMD ranges differ significantly between studies. Nosova et al. [21] reported that 5 day bed rest reduced mean FMD from 11 ± 3% to 9 ± 2%, whereas in a study by Benjamin et al. [22] FMD values of 3.3 (±3.0)% for women and 2.4 (±2.4)% for men were reported, while Gokce et al. [23] reported mean FMD values of 6.6 (±4,7)% in patients undergoing vascular surgery. The differences between the studies show that an individual value, without a reference or control, does not favour prognostic assessment and that FMD analysis is perhaps more valuable in the context of clinical studies than in clinical practice [24]. However, we sought to investigate the impact of both the absolute FMD value and the dichotomized FMD (threshold 10%) on our study endpoints. This threshold was proposed by Kuven et al. [17] and our analyis revealed that applying this threshold leads to a significant discrimination of the two groups regarding their absolute FMD values. Despite the fact that brachial artery reactivity has been associated with the prognosis of cardiovascular events and long-term outcome in previous studies [6, 25, 26], we could not prove a predictive effect for DAH30 or the occurrence of postoperative complications. However, in our study, the population of patients with non-cardiac surgery was different and the observation period was relatively short (30 days). We previously reported that low FMD correlated with longer lengths of ICU and hospital stay in patients having thoracic surgery, where the spectrum of operations included major lung surgery besides oesophagectomies [10]. Moreover, the comorbid burden was higher within these patients, indicating that FMD may not be suited for risk prediction in abdominal surgery or patients with relatively low comorbid burden. Lung surgery may be different in the context of lung capillary endothelial function being crucial for homeostasis, while its dysfunction can lead to highly life-threatening pathologies such as lung embolism, acute respiratory distress syndrome and pulmonal artery hypertension, all of which have previously been associated with reduced FMD [27–29].

Oesophagectomy was not found to be an independent predictor of postoperative outcome, when ASA status and duration of surgery were also regarded within the regression model. However, as we could demonstrate that established factors for impaired postoperative outcome including ASA status and duration of surgery are capable of predicting the primary and secondary endpoints, our data appears to be plausible.

Limitations to our study, however, include the fact that FMD measurements may be prone to a high variation between measurements, as vascular function is physiologically altered by many factors. Peretz et al. [30] have noted a deviation of 2.4% in repeated measurements using the upper arm occlusion technique compared to 1.2% using the forearm occlusion technique. Similar consideration should be given to patient medications that modulate vascular function. Corretti and Thijssen [15, 31] draw attention to the importance of stopping any medication with vascular influence for at least four half-lifes, as supported by ACE inhibitiors and statins reported to increase FMD values [32]. However, within our study population there were no differences among the intake of ACE inhibitors or statins between the groups of patients with normal versus reduced FMD, whilst AT1 antagonist intake was more frequent in individuals with reduced FMD. Modulation with such medications may explain the observed variability in correlation between FMD and well-established preoperative cardiovascular risk factors, but may highlight the potential use of serial FMD measurements to monitor response to such therapies. Consideration should also be given to having patients fast before testing and to refrain from smoking, caffeine or alcohol intake for at least 6 h. Testing should ideally be performed at the same time of day based on circadian rhythms. The challenge of ensuring these testing conditions is possible within a research context, but these factors renders the application of FMD poorly suited to point-of-care testing in daily clinical practice. Furthermore, ultrasound techniques may, despite a standardised guideline, vary between patients. Since the FMD analysis as part of the preoperative risk assessment is facing substantial practical limits, it is unlikely that it could be a helpful additional tool in routine clinical practice. The biggest hurdle of this technique is the fact that it is time consuming and therefore difficult to include in the preoperative evaluation of patients who are candidates for surgery. Furthermore, a validation of this technique to be a true predictor of increased perioperative risk has yet to be shown.

## Conclusion

This study found no additional predictive value of the FMD for DAH30 or the occurrence of postoperative complications determined by CCI and Clavien-Dindo score. Further investigation regarding these findings is needed, as it seems that FMD is dependent on the patients underlying disease state and thus homogenous groups of patients should be studied. FMD remains an interesting and potentially useful tool for risk assessment in cardiovascular diseases, however, its value in the perioperative context requires further investigation.

## Data Availability

The datasets used and/or analyzed during the current study are available from the corresponding author on reasonable request.

## Abbreviations

BART: Brachial artery reactivity testing
BMI: Body Mass Index
CCI: Comprehensive Complication Index
95% CI: 95% Confidence intervall
CD: Clavien-Dindo
DAH30: Days at Home within 30 days after surgery
FMD: Flow-mediated dilation
IQR: Interquartile range
MET: Metabolic equivalent of Task
OR: Odds ratio
rCRI: Revised Cardiac Risk Index
SORT: Surgical Outcome Risk Tool
